# Community Assessments for Mosquito Prevention and Control Experiences, Attitudes, and Practices — U.S. Virgin Islands, 2017 and 2018

**DOI:** 10.15585/mmwr.mm6822a3

**Published:** 2019-06-07

**Authors:** Krystal R. Seger, Joseph Roth, Amy H. Schnall, Brett R. Ellis, Esther M. Ellis

**Affiliations:** ^1^U.S. Virgin Islands Department of Health; ^2^Center for Preparedness and Response, CDC; ^3^National Center for Environmental Health, CDC.

*Aedes aegypti*, the mosquito that carries dengue, chikungunya, and Zika viruses, is present throughout the U.S. Virgin Islands (USVI). To reduce mosquitoborne disease transmission, the USVI Department of Health (VIDOH) is responsible for integrated mosquito management. During January 2016–January 2018, USVI experienced its first Zika outbreak, with most cases reported during January–December 2016, as well as two Category 5 hurricanes (Irma on St. Thomas/St. John on September 6, 2017, and Maria on St. Croix on September 19, 2017). The hurricanes severely damaged mosquito protection–related building structures (e.g., screens, roofs) and infrastructure (e.g., electricity, air conditioning) and might have created an environment more conducive to mosquito breeding. VIDOH, with requested technical assistance from CDC, conducted three Community Assessments for Public Health Emergency Response (CASPERs) to provide rapid community information at the household level. The three CASPERs were conducted to inform 1) the Zika outbreak response, 2) the hurricane response, and 3) the hurricane recovery. The CASPERs assessed mosquito prevention and control-related experiences, attitudes, and practices; household and environmental conditions associated with mosquito breeding, prevention, and control; and other nonmosquito-related information to inform outbreak and disaster response planning. Approximately 40% of households were very concerned about contracting Zika virus during the Zika outbreak and hurricane responses. Environmental conditions were reported to become more favorable for mosquito breeding between the Zika outbreak and hurricane response. Between 75%–80% of the community supported mosquito-spraying in all assessments. VIDOH used these data to support real-time outbreak and hurricane response planning. Mosquito prevention and control community assessments can provide rapid, actionable information to advise both mosquito education and control and emergency response and recovery efforts. The CASPER design can be used by vector control programs to enhance routine and response operations.

The Zika outbreak response CASPER was conducted during June 26–29, 2017, on the three main islands, St. Croix, St. Thomas, and St. John. The hurricane response CASPER was conducted in two geographically distinct districts (St. Croix on November 7–8, 2017, and St. Thomas/St. John on November 13–14, 2017) to account for the two hurricanes. The same questionnaire was used for both CASPERs, and the results from both locations were similar; therefore, they were considered and analyzed together as one CASPER. The hurricane recovery CASPER was conducted during February 26–March 1, 2018, on the three main islands.

The standard CASPER two-stage cluster sampling methodology was used to select a representative sample of interviewed households (*1*). The sampling frame was defined as all 43,214 occupied households within USVI, according to the 2010 U.S. Census. Using the Geographic Information Systems CASPER toolkit (*1*), 30 clusters were selected with probability of selection proportional to the number of households within each cluster. Interview teams were trained to select seven households from each of the selected clusters by systematic random sampling, with a goal of 210 interviews for each assessment. Teams made three attempts to contact one adult resident for an interview in each household before substituting another household.

The three 2-page CASPER questionnaires included the same or similar questions regarding mosquito prevention and control experiences, attitudes, and practices, including mosquito biting activity, repellent use, and household environmental characteristics. Response frequencies and percentages, including completion rates, with 95% confidence intervals (CIs) were calculated using Epi Info (version 7.2.2.2; CDC). Weighted frequencies and percentages based on probability of selection are reported, with weighted analysis only calculated for cells with ≥5 households (*1*). A preliminary report was presented to VIDOH within 5 days of completion of each assessment.

Teams conducted 201 of the target 210 interviews for the Zika outbreak response CASPER (95.7% completion rate; 62.2% of contacted households); 387 of the target 420 interviews for the hurricane response CASPER, including 195 on St. Croix (92.9% completion rate; 84.1% of contacted households) and 192 on St. Thomas/St. John (91.4% completion rate; 84.2% of contacted households); and 200 of the target 210 interviews for the hurricane recovery CASPER (95.2% completion rate; 81.3% of contacted households). The most represented household member age group in all three CASPERs was persons aged 18–64 years (80.8%, 75.0%, and 76.6% for the Zika outbreak response, the hurricane response, and the hurricane recovery CASPERs, respectively) followed by those aged ≥65 years (41.5% [Zika outbreak], 42.5% [hurricane response], and 42.2% [hurricane recovery]).

During the Zika outbreak response, 72.3% of households were very or somewhat concerned about contracting Zika virus, whereas 25.3% were not concerned; 78.7% were very or somewhat concerned about contracting other mosquitoborne diseases, including malaria, dengue, chikungunya, or yellow fever, and 17.8% were not concerned (Table 1). During the hurricane response, 87% of households noticed an increase in mosquito biting since the storms; however, only 61.5% were very or somewhat concerned about contracting Zika virus, 61.3% were concerned about contracting other mosquitoborne diseases, and 37.4% were not concerned. During hurricane recovery, 39.8% of households noticed an increase in mosquito biting during the preceding 4 weeks; approximately two thirds were very or somewhat concerned about contracting any mosquitoborne disease, and 32.7% were not concerned.

**TABLE 1:** Weighted household mosquitoborne disease concerns from the Community Assessments for Public Health Emergency Response (CASPERs) — U.S. Virgin Islands, 2017–2018

Observations and concerns	Zika outbreak response		Hurricane response		Hurricane recovery	
	June 2017 (n = 201)		November 2017 (n = 387*)		February 2018 (n = 200)	
	Estimate^†^	% of HH (95% CI)	Estimate^†^	% of HH (95% CI)	Estimate^†^	% of HH (95% CI)
**Noticed increase in mosquito biting in past 4 weeks^§^**						
Yes	—^§^	—^§^	37,617	87.0 (83.4–90.7)	17,203	39.8 (31.4–48.2)
Changed daily activities	—^§^	—^§^	23,469	63.3 (57.1–69.6)	9,967	58.6 (47.3–70.0)
Did not change activities	—^§^	—^§^	13,590	36.7 (30.4–42.9)	7,031	41.4 (30.0–52.7)
No	—^§^	—^§^	5,597	13.0 (9.3–16.6)	26,011	60.2 (51.8–68.6)
**Household current concern about contracting Zika virus^¶^**						
Very concerned	17,725	41.0 (31.4–50.6)	16,113	37.3 (32.3–42.3)	—^¶^	—^¶^
Somewhat concerned	13,540	31.3 (23.8–38.9)	10,438	24.2 (18.4–29.9)	—^¶^	—^¶^
Not concerned at all	10,961	25.3 (18.5–32.2)	16,192	37.5 (32.3–42.7)	—^¶^	—^¶^
Don’t know	—**	—**	471	1.1 (0.0–2.2)	—^¶^	—^¶^
**Household current concern about contracting other mosquitoborne diseases^¶^**						
Very concerned	21,216	49.1 (40.9–57.3)	16,137	37.3 (32.0–42.7)	—^¶^	—^¶^
Somewhat concerned	12,786	29.6 (21.6–37.6)	10,367	24.0 (18.2–29.8)	—^¶^	—^¶^
Dengue^††^	14,528	42.7 (34.7–50.8)	11,994	45.0 (36.2–53.8)	—^¶^	—^¶^
Chikungunya^††^	10,076	29.6 (22.0–37.3)	9,593	36.0 (28.6–43.4)	—^¶^	—^¶^
Malaria^††^	3,821	11.2 (6.6–15.9)	3,280	12.3 (8.2–16.4)	—^¶^	—^¶^
Yellow fever^††^	—**	—**	1,775	6.7 (2.7–10.6)	—^¶^	—^¶^
Other/Don’t know^††^	13,767	40.5 (30.9–50.0)	9,074	34.2 (26.1–42.3)	—^¶^	—^¶^
Not concerned at all	7,689	17.8 (10.9–24.7)	16,145	37.4 (31.4–43.3)	—^¶^	—^¶^
Don’t know	1,523	3.5 (0.8–6.2)	565	1.3 (0.1–2.5)	—^¶^	—^¶^
**Household current concern about contracting mosquitoborne diseases^¶^**						
Very concerned	—^¶^	—^¶^	—^¶^	—^¶^	16,764	38.8 (30.3–47.3)
Somewhat concerned	—^¶^	—^¶^	—^¶^	—^¶^	12,306	28.5 (20.9–36.1)
Zika^††^	—^¶^	—^¶^	—^¶^	—^¶^	13,640	46.9 (36.6–57.2)
Dengue^††^	—^¶^	—^¶^	—^¶^	—^¶^	12,789	44.0 (33.7–54.3)
Chikungunya^††^	—^¶^	—^¶^	—^¶^	—^¶^	8,643	29.7 (20.0–39.5)
Malaria^††^	—^¶^	—^¶^	—^¶^	—^¶^	5,803	20.0 (10.7–29.2)
Yellow fever^††^	—^¶^	—^¶^	—^¶^	—^¶^	3,018	10.4 (1.8–19.0)
Other/Don’t know^††^	—^¶^	—^¶^	—^¶^	—^¶^	6,568	22.6 (14.5–30.7)
Not concerned at all	—^¶^	—^¶^	—^¶^	—^¶^	14,144	32.7 (25.4–40.0)

**Abbreviations:** CI = confidence interval; HH = household.

* Two geographically distinct districts were used for the hurricane response CASPER, but the same questionnaire was used, and the presented results had no significant differences; therefore, they are considered and analyzed as one CASPER, resulting in the larger “n” than in the Zika outbreak response and hurricane recovery CASPERs.

^†^ Estimated number of U.S. Virgin Islands’ households.

^§^ Hurricane response CASPER asked “since the storms.” This question was not asked in the Zika outbreak response CASPER.

^¶^ Responses from the Zika outbreak and hurricane response CASPERs are not directly comparable to responses from the hurricane recovery CASPER because the questions were asked differently. Questions asked in the Zika outbreak and hurricane response CASPERs were “Currently, how concerned are you and members of your household about getting the Zika virus?” and “Currently, how concerned are you and members of your household about getting other diseases mosquitoes may carry?” The question asked in the hurricane recovery CASPER was “Currently, how concerned are you and members of your household about getting diseases mosquitoes may carry?” with a follow-up question for specific diseases.

** Number of responses was too few to be weighted.

^††^ Subcategories are a combination of both “very concerned” and “somewhat concerned.” Multiple responses were permitted.

Barriers to use of mosquito repellent differed between the Zika outbreak and hurricane responses (Table 2). During the Zika outbreak response, approximately half (49.0%) of households had no barriers to mosquito repellent use, although nearly a quarter (23.5%) did not like the feel or smell, and one in five (19.4%) was concerned about their health when using it; 3.9% said it was too expensive. During the hurricane response, a larger percentage (59.3%) had no barriers, and fewer did not like the feel or smell (12.5%) or were concerned about their health when using it (10.8%); more than twice as many (8.9%) said it was too expensive.

**TABLE 2:** Weighted household barriers to mosquito repellent use and household environmental characteristics from the Community Assessments for Public Health Emergency Response (CASPERs)* — U.S. Virgin Islands, 2017

Barriers and characteristics	Zika outbreak response		Hurricane response	
	June 2017 (n = 201)		November 2017 (n = 387^†^)	
	Estimate^§^	% of HH (95% CI)	Estimate^§^	% of HH (95% CI)
**Household barriers to mosquito repellent^¶^**				
Don’t like how it feels/smells	10,159	23.5 (18.0–29.0)	5,393	12.5 (8.9–16.1)
Concerned about health	8,396	19.4 (12.6–26.2)	4,681	10.8 (7.1–14.5)
Prefer natural remedies	4,637	10.7 (5.4–16.0)	4.760	11.0 (6.8–15.2)
Too expensive	1,681	3.9 (0.8–7.0)	3,854	8.9 (5.7–12.1)
Concerned for environment	1,399	3.2 (0.3–6.2)	1,904	4.4 (2.1–6.7)
No availability	—**	—**	2,444	5.7 (2.6–8.7)
Takes too much time	—**	—**	672	1.6 (0.0–3.2)
Other^††^	1,440	3.3 (0.4–6.2)	2,304	5.3 (2.0–8.6)
No barriers	21,195	49.0 (41.4–56.7)	25,642	59.3 (53.5–65.2)
**Household has the following^¶^:**				
Undamaged window screens	27,801	64.3 (54.7–74.0)	12,980	30.0 (24.1–36.0)
Undamaged door screens	17,238	39.9 (30.7–49.0)	9,813	22.7 (17.0–28.4)
Air conditioning	17,711	41.0 (31.5–50.4)	8,578	19.8 (15.0–24.7)
Objects that may collect rain	11,194	25.9 (19.5–32.3)	13,096	30.3 (23.7–36.9)
Abandoned buildings nearby	10,817	25.0 (15.5–34.5)	12,960	30.0 (22.7–37.3)
Uncovered water source	6,784	15.7 (9.4–22.0)	6,320	14.6 (10.6–18.7)
None of the above^§§^	5,055	11.7 (4.5–18.9)	10,762	24.9 (18.6–31.2)

**Abbreviations:** CI = confidence interval; HH = household.

* Questions were only asked during the Zika outbreak response CASPER and the hurricane response CASPER, and not for the hurricane recovery CASPER.

^†^ Two geographically distinct districts were used for the hurricane response CASPER, but the same questionnaire was used, and the presented results had no significant differences; therefore, they are considered and analyzed as one CASPER, resulting in the larger “n” than in the Zika outbreak response and hurricane recovery CASPERs.

^§^ Estimated number of U.S. Virgin Islands’ households.

^¶^ Multiple responses were permitted.

** Number of responses was too few to be weighted.

^††^ Includes too time-consuming, product not available, forgot, etc.

^§§^ Includes households that had both no sources for mosquito breeding and households with damaged screens and no air conditioning.

Reported environmental conditions became more favorable for mosquito breeding and exposure to mosquito bites between the Zika outbreak and hurricane responses. For example, the percentages of households with undamaged window screens, undamaged door screens, and air conditioning were 64.4%, 39.9%, and 41.0%, respectively, during the Zika outbreak response. These percentages declined to 30.0%, 22.7%, and 19.8% during the hurricane response.

Community support for VIDOH to spray for mosquitoes was similar during the Zika outbreak response and hurricane recovery (76.3% each) and the hurricane response (79.2%) (Table 3), although support for specific spray methods varied. Support for truck spraying increased from 63% of households during Zika outbreak response to 78.1% during hurricane response and returned to 63% during hurricane recovery. Outdoor backpack spraying was supported by only 29.6% of households during the Zika outbreak response, increasing to 44.8% during the hurricane response and to 61.9% during hurricane recovery. Aerial spraying was supported by 12.8% of households during Zika outbreak response, 28.8% during hurricane response, and 16.4% during hurricane recovery.

**TABLE 3:** Weighted household desired Department of Health mosquitoborne disease prevention and control actions from the Community Assessments for Public Health Emergency Response (CASPERs) — U.S. Virgin Islands (USVI), 2017–2018

Desired VIDOH prevention and control actions*^,†^	Zika outbreak response		Hurricane response		Hurricane recovery	
	June 2017 (n = 201)		November 2017 (n = 387^§^)		February 2018 (n = 200)	
	Estimate**^¶^**	% of HH (95% CI)	Estimate**^¶^**	% of HH (95% CI)	Estimate**^¶^**	% of HH (95% CI)
Spraying/Fogging (any)^†^	32,959	76.3 (69.2–83.3)	34,243	79.2 (75.4–83.1)	32,966	76.3 (70.7–81.9)
By truck	27,094	62.6 (55.3–70.1)	26,747	78.1 (73.4–82.8)	24,872	63.4 (56.5–70.4)
By hand (backpack)	12,779	29.6 (20.4–38.7)	15,358	44.8 (38.0–51.7)	24,286	61.9 (51.5–72.4)
By plane (aerial)	5,515	12.8 (6.5–19.1)	9,858	28.8 (22.3–35.2)	6,444	16.4 (10.5–22.4)
Other (e.g., unsure, “best way”)	3,190	7.4 (3.5–11.2)	2,834	8.3 (5.4–11.2)	—**	—**
Education	16,435	38.0 (27.8–48.2)	13,179	30.5 (23.6–37.4)	—*	—*
Inspection of property	10,563	24.4 (15.1–33.8)	9,759	22.6 (16.9–28.3)	—*	—*
Other^††^	5,961	13.8 (8.0–19.6)	6,491	15.0 (11.0–19.1)	—*	—*
Don’t know/None	1,440	3.3 (1.1–5.6)	3,011	7.0 (3.9–10.0)	—*	—*

**Abbreviations:** CI = confidence interval; HH = household; VIDOH = USVI Department of Health.

* Responses from the Zika outbreak and hurricane response CASPERs are not directly comparable to responses from the hurricane recovery CASPER because the questions were asked differently. Questions asked in the Zika outbreak and hurricane response CASPERs were “What actions do your HH members believe the health department should take to prevent mosquito diseases?” and “If spraying, which type(s) would you support?” The questions asked in the hurricane recovery CASPER was “Would your HH support any spraying for mosquitoes?” and “If yes, which type(s) would you support?”.

^†^ Multiple responses were permitted.

**^§^** Two geographically distinct districts were used for the hurricane response CASPER, but the same questionnaire was used, and the presented results had no significant differences; therefore, they are considered and analyzed as one CASPER, resulting in the larger “n” than in the Zika outbreak response and hurricane recovery CASPERs.

^¶^ Estimated number of USVI households.

** Number of responses was too few to be weighted.

^††^ Other includes property services, social services or assistances, material aid, etc.

## Discussion

These community assessments conducted during the Zika outbreak, hurricane responses, and hurricane recovery in USVI found that households were more concerned about contracting mosquitoborne diseases shortly after the Zika outbreak than during the hurricane response and hurricane recovery, even though reported mosquito biting activity increased, and environmental conditions were more favorable for mosquito breeding and exposure to bites following the hurricanes. In addition, although mosquitoborne diseases are endemic in USVI, and the population might be aware of the risk, households had concerns after the hurricanes that did not exist during the Zika outbreak, such as lack of shelter, clean water, and electricity (*2*). These differing levels of concern did not, however, change the community’s support for mosquito spraying, although support for specific spray methods varied.

VIDOH used the CASPER data to make real-time outbreak and hurricane response decisions to improve mosquito bite prevention, mosquito control, and community education. For example, because the percentage of households concerned about contracting mosquitoborne diseases declined after the hurricanes compared with during the Zika outbreak response, VIDOH hurricane response education campaigns prioritized household-level mosquito bite prevention. The differing levels of support for various spray methods were also recognized and considered during decision-making. For example, these data, along with unique environmental considerations, were used by the administration in place during the responses and recovery to determine backpack spraying to be the only acceptable option.

The CASPER is a useful tool for assessing mosquitoborne disease risk factors and creating immediately useable data to guide vector-related public health campaigns (*3*). According to CDC’s internal CASPER database (*4*), a limited number of CASPERs have been conducted that assess mosquito bite prevention- and control-related factors, such as knowledge of mosquitoborne diseases; ways to protect against mosquito bites; and how to identify, quantify, and manage potential mosquito breeding sites. Even fewer CASPERs have focused solely on mosquitoes. A CASPER in Long Beach, California, during a Zika outbreak identified the need for increased mosquito abatement (*5*). In two areas of Texas, CASPERs successfully assessed the prevalence of vectorborne disease risk factors and the communities’ knowledge of mosquito bite prevention and Zika virus (*6*,*7*). A CASPER conducted in American Samoa identified increased vector problems and the need for vector control after a tsunami (*8*).

Not only is CASPER an important tool for emergency response and recovery, it is also useful for collecting community public health information unrelated to an emergency (*4*,*9*). Vector control programs can use CASPERs during nonemergency situations to enhance and increase operation efficacy by evaluating the effectiveness of community campaigns and understanding community knowledge, attitudes, and practices.

The findings in this report are subject to at least three limitations. First, data generated from the CASPERs represent discrete points in time, which should be considered when interpreting the results to guide outbreak and hurricane response and recovery efforts. Second, the age distribution of the survey respondents is skewed, with a larger proportion of persons aged ≥65 years represented in the CASPERs than that reported by the U.S. Census; therefore, households without persons aged ≥65 years might be underrepresented. Finally, some questions were asked differently or not at all among the three CASPERs presented and are not directly comparable.

CASPERs that include mosquito prevention- and control-related questions are an important tool to inform both routine and response vector control operations and to understand how a community’s perceptions and behaviors might vary by adverse event and over time.

**Summary**

**What is already known about this topic?**

Integrated vector management is important to reduce mosquitoborne disease transmission. Community assessments are rarely used to inform mosquito management or understand related community perceptions.

**What is added by this report?**

Community assessments conducted in the U.S. Virgin Islands during the Zika outbreak response, hurricane response, and hurricane recovery found similar support for mosquito spraying, but support for specific spray methods varied. Concern about acquiring Zika decreased over time.

**What are the implications for public health practice?**

Mosquito prevention and control community assessment questions can provide rapid, actionable information to advise both community education and mosquito control in emergency response and recovery efforts. Assessments can also be used by vector control programs to enhance routine operations.
